# Unveiling the Crucial Role of Type IV Secretion System and Motility of *Helicobacter pylori* in IL-1β Production via NLRP3 Inflammasome Activation in Neutrophils

**DOI:** 10.3389/fimmu.2020.01121

**Published:** 2020-06-09

**Authors:** Ah-Ra Jang, Min-Jung Kang, Jeong-Ih Shin, Soon-Wook Kwon, Ji-Yeon Park, Jae-Hun Ahn, Tae-Sung Lee, Dong-Yeon Kim, Bo-Gwon Choi, Myoung-Won Seo, Soo-Jin Yang, Min-Kyoung Shin, Jong-Hwan Park

**Affiliations:** ^1^Laboratory Animal Medicine, College of Veterinary Medicine and Animal Medical Institute, Chonnam National University, Gwangju, South Korea; ^2^Department of Microbiology, School of Medicine, Gyeongsang National University, Jinju-si, South Korea; ^3^School of Bioresources and Bioscience, Chung-Ang University, Anseong, South Korea

**Keywords:** bacterial motility, *Helicobacter pylori*, IL-1β, neutrophils, type IV secretion system (T4SS)

## Abstract

*Helicobacter pylori* is a gram-negative, microaerophilic, and spiral-shaped bacterium and causes gastrointestinal diseases in human. IL-1β is a representative cytokine produced in innate immune cells and is considered to be a key factor in the development of gastrointestinal malignancies. However, the mechanism of IL-1β production by neutrophils during *H. pylori* infection is still unknown. We designed this study to identify host and bacterial factors involved in regulation of *H. pylori*-induced IL-1β production in neutrophils. We found that *H. pylori*-induced IL-1β production is abolished in NLRP3-, ASC-, and caspase-1/11-deficient neutrophils, suggesting essential role for NLRP3 inflammasome in IL-1β response against *H. pylori*. Host TLR2, but not TLR4 and Nod2, was also required for transcription of NLRP3 and IL-1β as well as secretion of IL-1β. *H. pylori* lacking *cagL*, a key component of the type IV secretion system (T4SS), induced less IL-1β production in neutrophils than did its isogenic WT strain, whereas *vacA* and *ureA* were dispensable. Moreover, T4SS was involved in caspase-1 activation and IL-1β maturation in *H. pylori*-infected neutrophils. We also found that FlaA is essential for *H. pylori*-mediated IL-1β production in neutrophils, but not dendritic cells. TLR5 and NLRC4 were not required for *H. pylori*-induced IL-1β production in neutrophils. Instead, bacterial motility is essential for the production of IL-1β in response to *H. pylori*. In conclusion, our study shows that host TLR2 and NLRP3 inflammasome and bacterial T4SS and motility are essential factors for IL-1β production by neutrophils in response to *H. pylori*.

## Introduction

*Helicobacter pylori* (*H. pylori*) is a gram-negative, microaerophilic, and spiral bacterium that colonizes the human gastric mucosa. More than 50% of the world's population are infected with the bacteria and the infection lasts a lifetime. *H. pylori* is the etiologic agent of gastrointestinal disorders that cause chronic gastritis, peptic ulcer, gastric adenocarcinoma, and gastric mucosa-associated lymphoid tissue (MALT) lymphoma (1). For this reason, it was classified by the World Health Organization as a class I carcinogen in 1994 (2).

Interleukin 1β (IL-1β) is considered to be a key factor correlated with development of gastric malignancies (3). Recently, polymorphisms of the *IL-1B* gene and IL-1 receptor antagonist (IL-1RN) have been revealed to be associated with *H. pylori*-related gastric cancer in the Chinese, Italian, and Indian population (4–6). Moreover, when infected with *H. felis*, IL-β-overexpressed transgenic mice display accelerated gastric inflammation development, metaplasia, and carcinoma (7). Huang et al. also showed that *H. pylori*-induced gastric inflammation and DNA methylation were reduced in IL-1R-deficient mice or by administration of IL-1 receptor antagonist (IL-1ra) (8). In addition, infiltration of innate immune cells, such as neutrophils and macrophages, and a multiplicity of gastric cancer induced by *H. pylori* infection were significantly reduced in IL-1β-deficient mice (9). Increased expression of IL-8, IL-1β, and COX-2 genes was also observed in patients with chronic gastritis infected with *H. pylori* compared with *H. pylori* negative patients (10). These findings suggest that IL-1β may play a crucial role in the development of *H. pylori*-induced gastric inflammation and cancer.

As a cytosolic multiprotein complex, NLRP3 inflammasome is a major inflammatory pathway that is activated in response to a variety of signals, including microbial infection and tissue damage (11). Activation of NLRP3 inflammasome is composed of two-step signals. First, the priming step is initiated by transcription of pro-IL-1β and NLRP3 through the activation of NF-κB and AP-1 by pattern-recognition receptors (PRRs) in response to microbial stimuli (12, 13). The second step involves NLRP3 inflammasome oligomerization, caspase-1 activation, cleavage of pro-IL-1β by caspase-1, and then secretion of the mature IL-1β. This step is induced by various molecules, such as ATP, reactive oxygen species (ROS), and monosodium urate (MSU) (14). Several studies have demonstrated that *H. pylori* activates the NLRP3 inflammasome in innate immune cells, including dendritic cells (DCs) and neutrophils (15–18).

Neutrophils play an important role in host defense against bacterial and fungal pathogens (19, 20). Despite the crucial role in innate immune response, several studies have reported that neutrophils might be involved in gastric-cancer development (21, 22). This concept has been supported by the observation that there was more neutrophils recruitment in gastric-cancer tissue than in the tissues surrounding gastric cancer (23). Furthermore, the higher number of neutrophils in gastric cancer is correlated with increased levels of IL-8 (23). In addition to the potential role of neutrophils in gastric-cancer development, a recent study has also shown that *H. pylori* T4SS induced production of IL-1β in human neutrophils in a NLRP3 inflammasome-dependent manner (17). However, the exact molecular mechanisms by which *H. pylori* bacterial factors regulate production of IL-1β in host neutrophils are not well-defined. Thus, in this study, we sought to identify both bacterial and host factors associated with IL-1β production in neutrophils in response to *H. pylori* infection.

## Materials and Methods

### Mice

We purchased wild type (WT), TLR2-, TLR4-, and NOD2- deficient mice on C57BL/6 background from the Jackson Laboratory (Bar Harbor, ME, USA). NLRP3-, Capase-1/11-, ASC-, and NLRC4- deficient mice were kindly provided by Prof. Gabriel Núñez (University of Michigan, USA). TLR5-deficient mice were gifts from Prof. Joon Haeng Rhee (Chonnam National University, Hwasun, Korea). We conducted all animal studies using protocols approved by the Institutional Animal Care and Use Committee of Chonnam National University (Approval No. CNU IACUC-YB-2018-85).

### Bacterial Strains and Culture Conditions

*Helicobacter pylori* P1WT and its isogenic mutants P1Δ*CagL*, P1Δ*FlaA*, P1Δ*UreA*, and P1Δ*VacA* have been described previously (24). Another mutant with FlaA deficiency was generated by allelic exchange in *H. pylori* 26695 strain, and details are provided in the Supplementary Material. The following clinical isolates from child patients were provided from Gyeongsang National University Hospital (GNUH), as the Branch of National Culture Collection for Pathogens (NCCP, Jinju, Korea): three motile strains, *H. pylori* 5356AC*, H. pylori* 4930AC*, H. pylori* 5049AC; two non-motile strains, *H. pylori* 4940A*, H. pylori* 4980AC. *H. pylori* 52WT (non-motile) and its mouse-adapted strain *H. pylori* 52P6 (six time-passaged) were also provided from Gyeongsang National University Hospital. We cultured all *H. pylori* strains on Brucella broth containing 10% fetal bovine serum (FBS; Corning costar, Corning NY, USA), 1 μg/ml nystatin (Sigma-Aldrich, St. Louis, MO, USA, Cat No. N3503), 5 μg/ml trimethoprim (Sigma-Aldrich, Cat No. T7883), and 10 μg/ml vancomycin (Sigma-Aldrich, catalog no. V2002) at 37°C under microaerobic conditions.

### Cell Culture and Bacterial Infection

We isolated thioglycollate-induced peritoneal neutrophils as previously described (25). Briefly, mouse peritoneal neutrophils were harvested after intraperitoneal injection of 2 ml of 4% thioglycollate broth (Sigma-Aldrich, Cat No. 70157). Four hours later, mice were injected intraperitoneally with 5 ml of PBS and peritoneal lavage was obtained twice. Red blood cells (RBCs) were lysed with cell lysis buffer. These collected peritoneal neutrophils were cultured in RPMI 1640 (Welgene, Gyeongsan, Gyeongsangbuk-do, Korea) containing 10% FBS in a 5% CO_2_ incubator at 37°C. To obtain of neutrophils derived from bone marrow, we isolated cells from femurs and tibias using density gradient cell separation protocol. Total bone marrow cells were overlaid on a two-layer gradient of HISTOPAQUE-1119 (density: 1.119 g/ml; Sigma-Aldrich, Cat No. 11191) and HISTOPAQUE-1077 (density: 1.077 g/ml; Sigma-Aldrich, Cat No. 10771) and centrifuged (2,000 rpm, 30 min) without braking. The collected cells in the interface were used. Bone-marrow neutrophils (BMNs) were resuspended in RPMI 1640 (Welgene) containing 10% FBS in a 5% CO_2_ incubator at 37°C. Purity of isolated neutrophils was confirmed by flow cytometry (Miltenyi Biotec, Bergisch Gladbach, Germany). Cells showing CD11b^+^ (FITC-conjugated anti-CD11b, BD Biosciences, San Jose, CA, USA, Cat No. 552850) and Gr-1^+^ (APC-conjugated anti-Gr-1, BD Biosciences, Cat No. 553129) were > 90% (Supplementary Figures 1A,B). Human leukemia cell line HL-60 was cultured in RPMI 1640 medium (Welgene) containing 10% FBS, 1% Penicillin/Streptomycin (P/S; Gibco, Grand Island, NY, USA), 2 mM L-glutamine (Gibco), and 25 mM HEPES (Gibco). To differentiate into neutrophil-like cells, we stimulated cells with 1.25% DMSO for 7 days in a 5% CO_2_ incubator at 37°C. These cells were seeded into 6-well or 48-well plates at a density of 2 × 10^6^ cells or 2 × 10^5^ cells, respectively, and incubated in a 5% CO_2_ incubator at 37°C. Subsequently, cells were infected with *H. pylori* at the indicated dose and time points.

### Evaluation of Cytokine Secretion by Bacteria Motility

To determine whether bacteria motility affected the secretion of IL-1β in peritoneal neutrophils, mild centrifugation (1,000 rpm, 10 min) was performed immediately after *H. pylori* P1WT and P1Δ*FlaA* (MOI 100) infection. At 24 h after infection, cell supernatant was collected for cytokine measurement.

### Reagent and Inhibitor Assay

To activate the NLRP3 inflammasome, peritoneal neutrophils and BMNs were treated with 100 ng/ml LPS (InvivoGen, San Diego, CA, USA, Cat No. tlrl-eblps) for 3 h followed by treatment with 5 mM adenosine 5′-triphosphate disodium salt hydrate (ATP, Sigma-Aldrich, Cat No. A2383) for 45 min. For inhibitor assay, HL-60 cells were infected with *H. pylori* P1WT (MOI 100) for 24 h with or without pretreatment with glyburide (Sigma-Aldrich, Cat No. G2539) and Ac-YVAD-CMK (Calbiochem, La. Jolla, CA, USA, Cat No. 400012) for 2 h at indicated concentrations.

### Measurement of Cytokines

We measured the concentrations of IL-1β (Cat No. DY401) and tumor necrosis factor alpha (TNF-α, Cat No. DY410) in culture supernatants of *H. pylori*-infected cells by using a commercial ELISA kit (R&D Systems, Minneapolis, MN, USA). To measure the levels of IL-18 in culture supernatant from *H. pylori*-infected peritoneal neutrophils, anti-mouse IL-18 antibody (MBL International, MA, USA, Cat No. D047-3) was coated on 384-well plate overnight at room temperature. The coated plate was washed three times with PBS containing 0.05% tween 20 (Sigma-Aldrich). After washed, the plate was incubated with 1% BSA for blocking for 1 h at room temperature. With three times washing between each step, the plate was further incubated with samples of culture supernatants for 2 h, the biotinylated-anti-mouse-IL-18 (MBL International, Cat No. D048-6) for 2 h, and the Streptavidin-HRP (R&D Systems) for 20 min at room temperature. Finally, the plate was washed and treated with the substrate solution (R&D Systems, Cat No. DY999). After the addition of stop solution, the optical density was measured at 450 nm.

### Immunoblotting

Peritoneal neutrophils and BMNs were seeded at density of 2 × 10^6^ cells/well in sterile 35 mm dishes and incubated overnight. These cells were infected with *H. pylori* (MOI 100) for 6 h. Culture supernatants and remaining cells were lysed by 1% Triton-X 100 (Sigma-Aldrich, Cat No. T9284) and a complete protease inhibitor cocktail (Roche, Mannheim, Germany, Cat No. 11-839-170-001). After centrifugation at 3,000 rpm for 5 min, we mixed the supernatant with SDS-PAGE sample loading buffer (5×). To detect target proteins, samples were separated by 15% SDS-PAGE and transferred to nitrocellulose (NC) membranes (GE Healthcare, Chicago, IL, USA, Cat No. 10600003). We probed membranes with primary antibodies against caspase-1 (AdipoGen Life Sciences, San Diego, CA, USA, Cat No. AG-20B-0042), IL-1β (R&D Systems, Cat No. AF-401-NA), and β-actin (Santa Cruz Biotechnology, Dallas, TX, USA, Cat No. sc-47778). After immunoblotting with goat anti-mouse IgG (H+L) secondary antibody, HRP (Thermo Fisher Scientific, MA, USA, Cat No. 31430) or goat anti-rabbit IgG (H+L) secondary antibody, HRP (Thermo Fisher Scientific, Cat No. 31460), we detected proteins using Clarity^TM^ Western ECL Substrate (Bio-Rad, Hercules, CA, USA, Cat No. BR170-5061).

### Real-Time Quantitative PCR (qPCR)

RNAs from *H. pylori* infected cells were extracted using easy-BLUE^TM^ Total RNA Extraction Kit (Intron Biotechnology, Seongnam, Korea) and cDNA synthesis was done using ReverTra Ace® qPCR RT Master Mix (TOYOBO Bio-Technology, Osaka, Japan) according to the manufacturer's instructions. qPCR was performed by the CFX Connect^TM^ Real-time PCR Detection System (Bio-Rad, Hercules, CA, USA) using 2x PCRBIO SyGreen Blue Mix Lo-ROS according to the manufacturer's instructions (Bio-D Co., Ltd., Hull, UK). GAPDH was used for normalization. The primers used for qPCR were as follows:

mNLRP3 forward: 5′-ATGGTATGCCAGGAGGACAG-3′;
mNLRP3 reverse: 5′-ATGCTCCTTGACCAGTTGGA-3′;
mIL-1β forward: 5′-GATCCACACTCTCCAGCTGCA-3′;
mIL-1β reverse: 5′-CAACCAACAAGTGATATTCTCCATG-3′;
mGAPDH forward: 5′-CGACTTCAACAGCAACTCCCACTCTTCC-3′;
mGAPDH reverse: 5′-TGGGTGGTCCAGGGTTTCTTACTCCTT-3′.

### Statistical Analysis

Statistical significance of differences among groups was found by using two-tailed Student's *t*-test or the one- or two-way analysis of variance (ANOVA) followed by Bonferroni post-tests. We calculated all statistics using GraphPad Prism version 5.01 (GraphPad Software, San Diego, CA, USA). Values of *P* < 0.05 were considered statistically significant.

## Results

### *H. pylori* Induces IL-1β Production in Neutrophils by Activating NLRP3 Inflammasome by Stimulating Various Danger Signals

**Figure 1:**
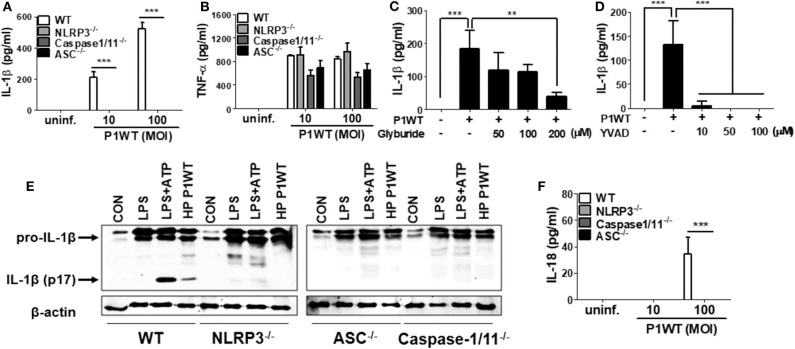
*H. pylori* mediates production of IL-1β in NLRP3 inflammasome-dependent manner in neutrophils. WT, and NLRP3-, Caspase1/11-, and ASC-deficient mouse peritoneal neutrophils were infected with *H. pylori* P1WT (MOI 100) for 24 h **(A,B,F)**. The concentration of IL-1β, TNF-α, and IL-18 in supernatant was measured by ELISA. HL-60 cells were pretreated without or with glyburide **(C)** or Ac-YVAD-CMK **(D)** at the indicated concentrations for 2 h and subsequently infected with *H. pylori* P1WT (MOI 100) for 24 h. The concentration of IL-1β in supernatant was measured by ELISA. We infected peritoneal neutrophils from WT and NLRP3-, ASC-, and Caspase1/11-deficient mice with *H. pylori* P1WT (MOI 100) for 6 h **(E)**. We used culture supernatants and cell lysates to detect immature and cleaved forms of IL-1β by Immunoblotting. Antibody against β-actin was used as a loading control. Results are presented as mean ± SD. **P < 0.01 and ***P < 0.001.

NLRP3 inflammasome has been known to be required for caspase-1 activation and IL-1β maturation in DCs in response to *H. pylori* (15, 16, 18). Therefore, we first investigated whether NLRP3 inflammasome is also involved in *H. pylori*-induced IL-1β maturation in neutrophils. As shown in Figure 1A, *H. pylori* P1WT induced IL-1β secretion in peritoneal neutrophils isolated from WT mice, whereas the production of IL-1β was abolished in NLRP3-, caspase-1/11-, and ASC-deficient neutrophils. Unlike the induction of IL-1β, *H. pylori*-induced TNF-α production was not affected by the same genetic deficiencies (Figure 1B). To confirm these data in a human-cell system, HL-60 cells, a human promyelocytic leukemia cell line, was differentiated to neutrophil-like cells at the presence of DMSO as previously described (26). As expected, *H. pylori* P1WT could induce IL-1β production in the differentiated HL-60 cells, which was reduced by the presence of glyburide (a NLRP3 inflammasome inhibitor) or Ac-YVAD-CMK (a caspase-1 inhibitor) in a dose-dependent manner (Figures 1C,D). Next, we did Western-blot analysis to detect cleaved IL-1β as a mature form. *H. pylori* P1WT led to cleavage of IL-1β in WT neutrophils, but not in cells from NLRP3-, caspase-1/11-, and ASC-deficient mice (Figure 1E, Supplementary Figure 2). These findings suggest that the NLRP3/ASC/caspase-1 axis is essential for *H. pylori*-induced IL-1β production in neutrophils. Furthermore, we observed that the NLRP3, caspase-1, and ASC were also required for IL-18 production in response to *H. pylori* in neutrophils (Figure 1F).

Various signals, such as extracellular ATP, K^+^ efflux, reactive oxygen species (ROS) generation, and cathepsin B release from lysosomes, mediates activation of NLRP3 inflammasome (14). To find out whether these stimuli are involved in *H. pylori*-induced IL-1β production in neutrophils, we carried out inhibitor assays. Oxidized ATP (oxATP) (a P2X7R antagonist) and extracellular addition of KCl reduced IL-1β production in *H. pylori* P1WT-infected neutrophils in a dose-dependent manner, and the cytokine production was completely abolished at a high concentration of those inhibitors, whereas TNF-α level was slightly influenced (Supplementary Figures 3A–D). We also observed that the detectable level of LDH was released even in untreated control cells (Supplementary Figure 4). The LDH release was increased by *H. pylori* infection, which was decreased by oxATP (Supplementary Figure 4), suggesting that dying or dead cells can be a source of extracellular ATP. A ROS inhibitor NAC reduced both IL-1β and TNF-α at 20 mM concentration (Supplementary Figures 3E,F). The IL-1β production was significantly inhibited by CA074Me, a cathepsin B inhibitor, even at a low concentration of 5 μM, which did not influence TNF-α level (Supplementary Figures 3G,H). These findings suggest that various extracellular signals, such as ATP/P2X7R, potassium efflux, ROS generation, and cathepsin B, may contribute to *H. pylori*-induced activation of the NLRP3 inflammasome pathway in neutrophils.

### TLR2, but Not Nod2 and TLR4, Is Required for Expression of IL-1β Gene and Secretion of IL-1β in *H. pylori*-Infected Neutrophils

**Figure 2:**
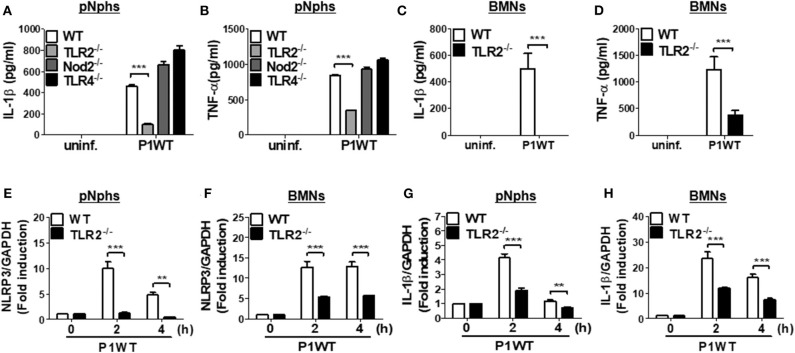
TLR2, but not NOD2 and TLR4, is involved in *H. pylori* recognition in mouse neutrophils. WT and TLR2-, NOD2-, and TLR4-deficient peritoneal neutrophils **(A,B)** and BMNs **(C,D)** from WT and TLR2-deficient mice were infected with P1WT (MOI 100) for 24 h. The concentration of IL-1β **(A,C)** and TNF-α **(B,D)** in the supernatant was measured by ELISA. We infected WT and TLR2-deficient peritoneal neutrophils or BMNs with *H. pylori* P1WT (MOI 100) at indicated times **(E–H)**. Gene expressions of NLRP3 **(E,F)** and IL-1β **(G,H)** were evaluated by real-time PCR. Results are presented as mean ± SD. **P < 0.01 and ***P < 0.001.

Previously, TLR2 and Nod2 have been shown to be involved in *H. pylori*-mediated gene expression of NLRP3 and IL-1β in DCs (15). Moreover, *H. pylori* upregulates TLR4 expression in human gastric epithelial cells (27). We therefore explored whether such patterns of recognition mediate IL-1β production in neutrophils in response to *H. pylori* infection. TLR2 deficiency led to impaired production of both TNF-α and IL-1β in *H. pylori* P1WT-infected peritoneal neutrophils derived from mice (Figures 2A,B). In contrast, Nod2 and TLR4-deficient cells produced a comparable level of IL-1β and TNF-α (Figures 2A,B). Consistently, *H. pylori*-induced production of IL-1β and TNF-α was impaired in TLR2-deficient bone marrow-derived neutrophils (BMNs) (Figures 2C,D), while the deficiency of Nod2 and TLR4 did not affect the two cytokines production (Supplementary Figures 5A,B). To find out whether TLR2 contributes to priming of NLRP3 and IL-1β (signal 1), we evaluated their gene expression by RT-PCR. As shown by the results in Figures 2E–H, we observed increased expression of genes for NLRP3 and IL-1β in WT peritoneal neutrophils and BMNs, whereas we observed significantly lower levels of NLRP3 and IL-1β gene transcription in TLR2-deficient cells (Figures 2E–H). Collectively, these data indicate that TLR2 is a central innate receptor for regulating NLRP3 inflammasome priming (signal) in *H. pylori*-infected neutrophils.

### *H. pylori* Type IV Secretion System Is Required for IL-1β Secretion Through Caspase-1 Activation and IL-1β Processing in Neutrophils

**Figure 3:**
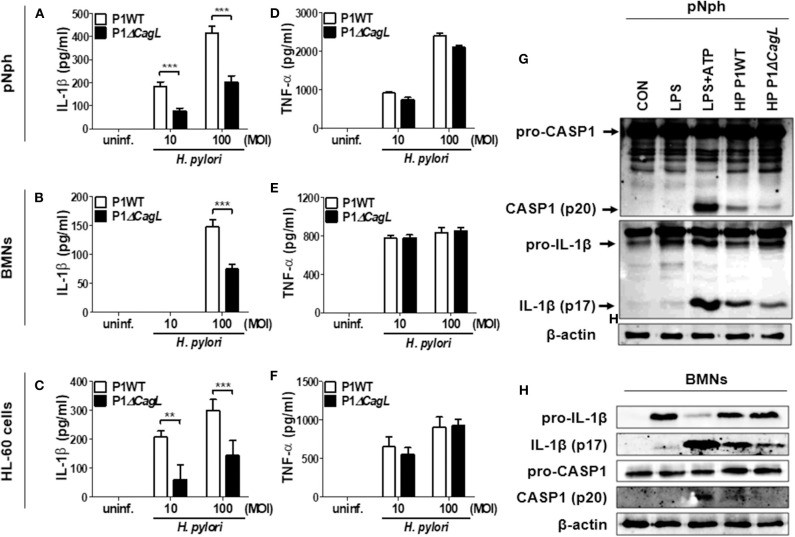
*H. pylori* T4SS induces production of IL-1β in neutrophils. Peritoneal neutrophils **(A,D)**, BMNs **(B,E)**, and HL-60 cells **(C,F)** were infected with *H. pylori* P1WT and isogenic mutant deficient in cagL (MOI 10 and 100) for 24 h. The concentration of IL-1β **(A–C)** and TNF-α **(D–F)** in supernatant was measured by ELISA. Results are presented as mean ± SD. **P < 0.01 and ***P < 0.001. Peritoneal neutrophils **(G)** and BMNs **(H)** were infected with P1WT and Δ*cagL* (MOI 100) for 6 h. We used culture supernatants and cell lysates to detect immature and cleaved forms of caspase-1 and IL-1β by Immunoblotting **(G,H)**. Antibody against β-actin was used as a loading control.

A previous report suggested that *H. pylori* uses a type IV secretion system (T4SS) to induce pro-IL-1β expression in DCs (15). In contrast, a recent study indicated that the *H. pylori* T4SS is dispensable for IL-1β production in human neutrophils (17). To assess the role of T4SS in production of IL-1β, we infected peritoneal neutrophils, BMNs, and the differentiated HL-60 cells with *H. pylori* P1WT and its isogenic mutant P1Δ*cagL*, in which T4SS is non-functional (28). As expected, we observed impaired production of IL-1β and IL-18 production in peritoneal neutrophils infected with P1Δ*cagL* (Figure 3A, Supplementary Figure 6). Consistently, *cagL* deficiency reduced the production of IL-1β in both BMNs and human neutrophil-like HL-60 cells (Figures 3B,C). In contrast to the production of IL-1β, the *cagL* mutant induced a comparable level of TNF-α in each cell type to its isogenic WT strain (Figures 3D–F). Western blot analysis revealed that cleavage of caspase-1 and IL-1β was reduced in P1Δ*cagL*-treated neutrophils more than in cells infected with P1WT (Figures 3G,H, Supplementary Figures 7A,B). There was no difference in expression level of the pro-form of IL-1β and casapse-1 between P1WT- and Δ*cagL*-infected neutrophils (Supplementary Figure 8). In addition, cagL deficiency did not influence the gene expression of NLRP3 and IL-1β in either peritoneal neutrophils or BMNs (Supplementary Figures 9A–D). These findings demonstrated that bacterial T4SS is involved in regulation of NLRP3 inflammasome activation (signal 2) rather than priming (signal 1) in host neutrophils in response to *H. pylori* infection.

### Deficiency of FlaA, but Not of UreA and VacA, Distinctly Leads to Impaired IL-1β Secretion in *H. pylori*-Infected Neutrophils

**Figure 4:**
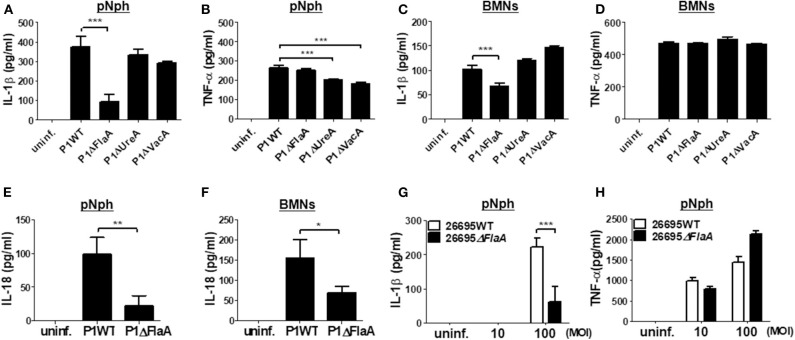
*H. pylori* flagellin is important for IL-1β production in mouse neutrophils. Peritoneal neutrophils **(A,B,E,G,H)** and BMNs **(C,D,F)** were infected with indicated bacteria (MOI 100 in A-F) for 24 h. We measured the concentration of IL-1β **(A,C,G)**, TNF-α **(B,D,H)**, and IL-18 **(E,F)** in the supernatant by ELISA. Results are presented as mean ± SD. *P < 0.05; **P < 0.01; and ***P < 0.001.

It has been reported that the *H. pylori* virulence factors, such as VacA and UreA, are involved in IL-1β production and NLRP3 inflammasome activation in DCs (16, 18). Bacterial flagellin has also been proposed to be involved in IL-1β processing via NLRC4 inflammasome activation (29). Therefore, we investigated whether such bacterial factors are required for *H. pylori*-induced IL-1β production in neutrophils. As shown in Figures 4A,B, both UreA and VacA mutant strains exhibited a similar level of IL-1β to the isogenic WT strain in peritoneal neutrophils, whereas TNF-α induction was slightly decreased in the two mutant strains (Figures 4A,B). Unexpectedly, IL-1β production was significantly lower in P1Δ*FlaA*-treated neutrophils than in cells infected with the P1WT strain, whereas levels of TNF-α were comparable between the two strains (Figures 4A,B). In BMNs, the FlaA deficiency also impaired the production of IL-1β, but not TNF-α (Figures 4C,D). Neither UreA nor VacA affected IL-1β and TNF-α production in BMNs in response to *H. pylori* (Figures 4C,D). Production of IL-18 was also impaired in both peritoneal neutrophils and BMNs infected with FlaA mutant (P1Δ*FlaA*) as compared with P1WT strain (Figures 4E,F). To confirm these data in a different *H. pylori* genetic background strain, we infected peritoneal neutrophils with *H. pylori* 26695WT and its isogenic mutant 26695Δ*FlaA*. In agreement with the data generated with the P1WT strain, IL-1β level was significantly lower in 26695Δ*FlaA*-treated cells than in ones treated with the 26695WT strain, whereas the TNF-α level was not affected by deletion of the FlaA gene (Figures 4G,H). To find out whether FlaA is required for IL-1β production in DCs, as DCs are previously known to be able to produce IL-1β in response to *H. pylori* (15), we infected BMDCs with *H. pylori* P1WT and P1Δ*FlaA*. As shown in Supplementary Figure 10, the P1WT and P1Δ*FlaA* strains displayed comparable levels of IL-1β secretion in DCs. These results indicated that *H. pylori* FlaA distinctly contributes to production of IL-1β in neutrophils, but not in DCs.

### FlaA Contributes to Both Priming and Inflammasome Activation of NLRP3 in Neutrophils in Response to *H. pylori*

**Figure 5:**
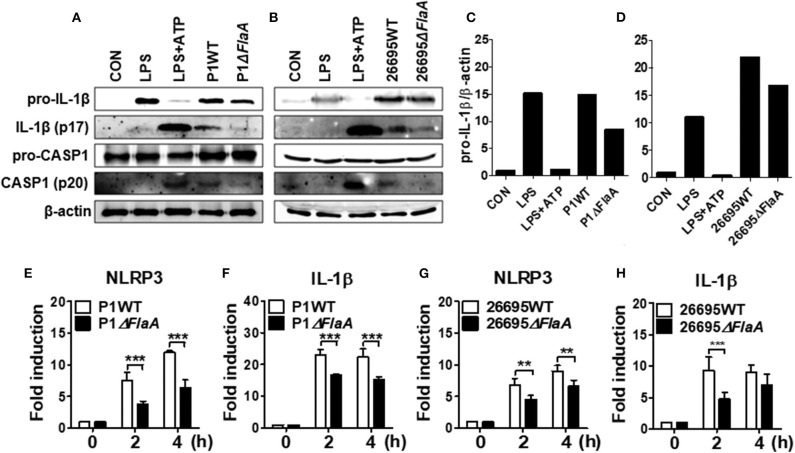
*H. pylori* flagellin induces activation of IL-1β and caspase-1 in response to *H. pylori* in mouse neutrophils. BMNs were infected with *H. pylori* P1WT and Δ*flaA* **(A,C)** or 26695WT and Δ*flaA* **(B,D)** (MOI 100) for 6 h. We used culture supernatants and cell lysates to detect immature and cleaved forms of caspase-1 and IL-1β by Immunoblotting **(A–D)**. Antibody against β-actin was used as a loading control. Peritoneal neutrophils **(E,F)** and BMNs **(G,H)** were infected with *H. pylori* P1WT (MOI 100) at indicated time. We evaluated gene expression of NLRP3 **(E,G)** and IL-1β **(F,H)** by real-time PCR. Results are presented as mean ± SD. **P < 0.01 and ***P < 0.001.

We next explored whether FlaA-induced production of IL-1β is mediated via activation of NLRP3 inflammasome in neutrophils. *H. pylori* P1WT induced strong expression of pro-IL-1β protein, which was slightly reduced in the cells treated with P1Δ*FlaA* (Figures 5A,C, Supplementary Figures 11A, 12A,C). P1WT-infected BMNs also released a significantly higher level of cleaved IL-1β and caspase-1 (mature form) than did BMNs infected with the P1Δ*FlaA* strain (Figure 5A, Supplementary Figure 11A). These results were also confirmed in the experiment using peritoneal neutrophils (Supplementary Figures 12A,C). Similar to the P1Δ*FlaA* strain, FlaA deficiency in the 26695 background also led to reduced cleavage of IL-1β and caspase-1 and less pro IL-1β expression (Figures 5B,D, Supplementary Figures 11B, 12B,D). In addition, expression of NLRP3 and IL-1β genes was significantly lower in P1Δ*FlaA*-treated BMNs P1WT-treated cells (Figures 5E,F). Consistently, NLRP3 gene expression was reduced in BMNs in response to FlaA deficiency in the *H. pylori* 26695 strain (Figure 5G). Moreover, gene expression of IL-1β was also decreased in 26695Δ*FlaA*-treated cells at 2 h after infection (Figure 5H). These data indicate that *H. pylori* flagellin is involved in regulation of both priming and activation of NLRP3 inflammasome in neutrophils.

### Bacterial Motility, but Not Host TLR5 and NLRC4, Is Required for *H. pylori*-Mediated IL-1β Production in Neutrophils

**Figure 6:**
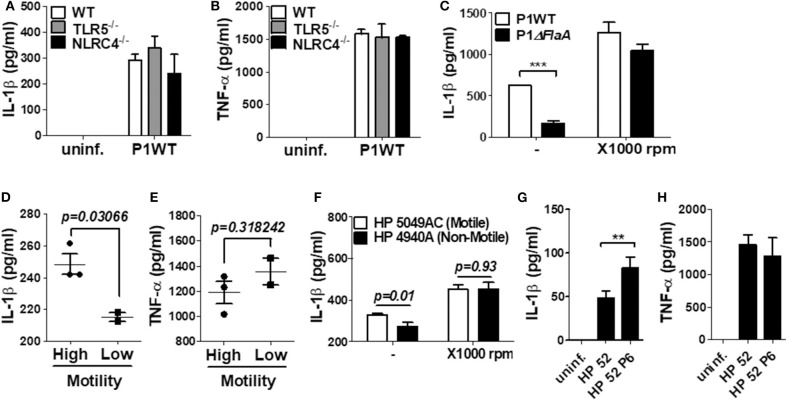
The production of IL-1β is regulated by *H. pylori* motility of flagellin, but not TLR5 and NLRC4 in mouse neutrophils. WT and TLR5-, and NLRC4-deficient peritoneal neutrophils were infected with *H. pylori* P1WT (MOI 100) for 24 h **(A,B)**. To promote the contact between cells and *H. pylori*, we used mild centrifuging (1,000 rpm, 10 min) **(C,F)**. Clinical isolates of *H. pylori* with or without motility were infected in peritoneal neutrophils **(D–F)**. *H. pylori* 52 and mouse-adapted bacteria (passage 6) were infected in peritoneal neutrophils **(G,H)**. The concentration of IL-1β **(A,C,D,F,G)** and TNF-α **(B,E,H)** in supernatant was measured by ELISA. Results are presented as mean ± SD. **P < 0.01 and ***P < 0.001, two-tailed Student's *t*-test.

TLR5 and NLRC4 are dual sensors for bacterial flagellin at the cell surface and cytosol in host cells (28). Flagellin recognition by TLR5 leads to NF-κB activation, whereas NLRC4 is responsible for inflammasome activation. Since *H. pylori* FlaA was essential for IL-1β gene expression and maturation in neutrophils, we tried to find out whether TLR5 and NLRC4 are involved in *H. pylori*-mediated IL-1β production. As shown by the results in Figures 6A,B, WT neutrophils and neutrophils deficient of TLR5 or NLRC4 produced a comparable level of IL-1β and TNF-α in response to *H. pylori*, suggesting that *H. pylori* FlaA regulates IL-1β production in neutrophils in a TLR5- and NLRC4-independent manner. Because bacterial flagella are essential for motility, we next explored whether motility is essential for *H. pylori*-induced IL-1β production in neutrophils. In our *in vitro* system, we cultured neutrophils in a floating state. Therefore, we centrifuged the cells after bacterial infection to increase the contact between *H. pylori* and neutrophils. As shown in Figure 6C, the centrifuging increased IL-1β production and abolished the difference of IL-1β production by WT and Δ*FlaA* mutant, suggesting that motility may be involved in *H. pylori*-induced IL-1β production in neutrophils. We further investigated whether different levels of motility in clinical isolates of *H. pylori* correlate with the amount of IL-1β production in neutrophils. Five clinical isolates of *H. pylori* were divided into two groups based on the results from a bacterial motility assay (Supplementary Figure 13): a low motility group of two strains (4940A and 4980AC) and a high motility group with three strains (4930AC, 5049AC, and 5356AC). *H. pylori* strains with high motility induced increased levels of IL-1β in neutrophils vs. the strains with low motility (Figure 6D), whereas TNF-α level was not different between the two groups of strains (Figure 6E). When 5049AC and 4940A strains were used as a representative one for motile and non-motile strain, the difference of IL-1β production was also abolished by the centrifugation (Figure 6F). Furthermore, *H. pylori* 52 strain originally displayed a low motility phenotype, but it obtained high motility phenotype (HP 52 P6) after six passages in mice (data not shown). Interestingly, the passaged *H. pylori* strain induced a significantly higher level of IL-1β in neutrophils than did the original strain with low motility (Figure 6G). The two strains produced comparable level of TNF-α (Figure 6H). At last, we tried to confirm the involvement of cagL and FlaA in the production of IL-1β using neutrophils purified from human blood. Purity of the isolated neutrophils were confirmed by Flow cytometry and cytospin (Supplementary Figures 14A,B). Consistently, cagL and FlaA deficiencies resulted in lower production of IL-1β, but not TNF-α, in human neutrophils (Supplementary Figures 14C,D).

## Discussion

IL-1β is considered to be a central factor for gastric malignancy, as is supported by evidence that *IL-1B* gene polymorphism increases the risk of gastric cancer (30, 31) and overexpression of IL-1β leads to gastric inflammation and cancers in mice (7, 9). It has been known that *H. pylori* leads to IL-1β production in DCs through an NLRP3-dependent pathway, although there were controversies about *in vivo* role of NLRP3 in the bacterial clearance in the stomach of mice (15, 16, 18). Neutrophils are key innate immune cells that are involved in *H. pylori*-mediated gastric inflammation (32). Therefore, it is conceivable that both bacterial and host factors may play crucial roles in the production of IL-1β in neutrophils. However, only limited information is available on the precise molecular mechanisms involved in *H. pylori*-induced IL-1β production in neutrophils.

Recently, Perez-Figueroa et al. reported that *H. pylori* induces IL-1β production in human neutrophils in TLR2- and TLR4-independent manner (17). They also demonstrated that *H. pylori* increases the expression of NLRP3 inflammasome components and inhibitors for NLRP3 and caspase-1 reduces the production of IL-1β (17). Moreover, in contrast to the important role of T4SS in DCs, T4SS was dispensable for *H. pylori*-induced IL-1β production in human neutrophils (17). However, some aspects are inconsistent with our current study. In this study, we identified that NLRP3 inflammasome is a key host factor in neutrophils for production of IL-1β in response to *H. pylori* by showing that secretion and cleavage of IL-1β were abolished in NLRP3-, caspase-1/11-, and ASC-deficient neutrophils. However, unlike the previous report by Perez-Figueroa et al. (17), our data suggested that TLR2 is required for *H. pylori*-induced IL-1β production in both peritoneal neutrophils and BMNs. Expression of NLRP3 and IL-1β genes was also reduced in TLR2-deficient neutrophils. In *H. pylori*-infected DCs, TLR2 has been known to mediate IL-1β production by regulating NLRP3 and pro IL-1β expression (signal 1) (15, 16). Furthermore, TLR2 contributes to NF-κB activation and chemokine expression in gastric epithelial cells in response to *H. pylori* (33). Nevertheless, a TLR2-independent pathway should be considered, as detectable levels of IL-1β and TNF-α were secreted in TLR2-deficient neutrophils. Although TNF-α is also responsible for a TLR-independent priming of NLRP3 inflammasome (34), its role in TLR2-independent production of IL-1β is likely limited, since there was already a significant difference in the gene expression of NLRP3 and IL-1β between WT and TLR2-deficient neutrophils within 2 h of *H. pylori* infection. Signaling of other pattern recognition receptors may lead to the priming of NLRP3 inflammasome in response to *H. pylori* in neutrophils. In addition, it seems to be necessary to measure the induction levels of NLRP3 or IL-1β expression in the human neutrophils prepared in the reports by Perez-Figueroa et al., because TLR2 may be dispensable if the cells are primed sufficiently.

Several bacterial factors including T4SS, vacuolating toxin, and urease contribute to IL-1β production in response to *H. pylori* in BMDCs (15, 16, 18). Isogenic mutants lacking both *ureA* and *ureB* genes failed to activate caspase-1, whereas transcription of IL-1β was unaffected (16). The *vacA* mutant also led to less production of IL-1β and impaired caspase-1 activation in LPS-primed BMDCs (18). There is still a controversy on the precise role of T4SS in regulation of IL-1β production in DCs. Kim et al. reported that the cagL mutant produced level of IL-1β similar to that of the isogenic WT strain in LPS-primed DCs, in which NLRP3 and IL-1β are sufficiently induced, whereas mRNA expression of IL-1β and its secretion were reduced in cagL mutant-treated cells in the unprimed condition (15), suggesting that T4SS is essential for the priming step. In contrast, Semper et al. showed that a mutant lacking cagE produced less IL-1β in LPS-primed DCs (18). In the present study, we demonstrated that T4SS, but not VacA and UreA, is required for *H. pylori*-induced IL-1β production in neutrophils. In *H. pylori*-infected neutrophils, T4SS seems to regulate signal 2 of inflammasome activation rather than priming of NLRP3 and pro IL-1β, because cagL deficiency did not influence protein expression of pro IL-1β or gene expression of NLRP3 and IL-1β. Our current results are inconsistent with the previous report that suggested a dispensable role of T4SS in *H. pylori*-induced production of IL-1β (17). In the previous study, the experimental design apparently was flawed by using the *H. pylori* 26695 strain as the WT strain and the virD4 mutant originating from a different genetic background, *H. pylori* G27 strain (17). G27 and 26695 strains differ in many aspects including salt sensitivity, the expression level of Lewis antigens, and resistance to acyl-lysyl oligomers (35–37). Because the ability to produce IL-1β can also differ among bacterial strains, this should be confirmed by using isogenic set of *H. pylori* strains. Furthermore, differences in origin of used cells should be considered. In most experiments, we used thioglycollate-elicited neutrophils, BMNs, and HL-60 cells, while in a study by Perez-Figueroa et al. (17), neutrophils purified from healthy donor blood were used. Cellular responses are likely to be different among these types of cells and, since cells purified from mice or humans are not entirely pure neutrophils, different compositions of contaminated cells can also affect results.

It is remarkable that the FlaA mutant led to less production of IL-1β in response to *H. pylori* in neutrophils, as was confirmed by using two sets of *H. pylori* strains, P1 and 26695. This seems to be specific in neutrophils, because FlaA deficiency did not influence IL-1β production in BMDCs. In addition, we showed that FlaA was required for gene transcription of NLRP3 and IL-1β and cleavage of caspase-1. TLR5 and NLRC4 are two central host receptors for the recognition of bacterial flagellin. TLR5 sensing of flagellin triggers NF-κB activation, followed by the production of pro-inflammatory cytokines (38). Bacterial flagellin can induce IL-1β production in innate immune cells through a NAIP-NLRC4-dependent pathway (39). However, in this study, both TLR5- and NLRC4-deficient neutrophils could produce a comparable level of IL-1β in response to *H. pylori*. In fact, it is known that *H. pylori* FlaA leads to weak activation of TLR5, and its purified flagellin fails to induce IL-8 production and p38 MAPK activation in gastric epithelial cells (40). Moreover, in contrast to *Salmonella typhimurium, H. pylori* flagellin could not induce activation of caspase-1 and production of IL-18 and LDH in macrophages, although it induced NLRC4 Ser533 phosphorylation (41). These findings indicate that *H. pylori* flagellin may contribute to production of IL-1β in neutrophils via a TLR5- and NLRC4-independent pathway. Instead, our results suggested that bacterial motility is essential for the production of IL-1β in response to *H. pylori*, as is supported by evidence that centrifuging that enhances bacteria to cell contact abolished the difference of IL-1β level produced by *H. pylori* P1WT and isogenic FlaA mutants and that clinical isolates with high motility produced more IL-1β than those with low motility. Since this phenomenon was seen only in neutrophils, but not BMDCs, a further study should be done to clarify the cell-type specific role of *H. pylori* flagellin in production of IL-1β. Additionally, our results showed that the bacterial motility in neutrophils is involved in both priming and activating NLRP3. It can be suggested as a mechanism, that *H. pylori* motility facilitates cell-to-cell contact, which may enhance NLRP3 priming by activating TLR2 and promote T4SS-mediated NLRP3 activation.

In conclusion, we shows here that *H. pylori* T4SS and flagellin are essential for IL-1β production in neutrophils. TLR2 and NLRP3 inflammasome are central host factors to regulate neutrophil production of IL-1β in response to *H. pylori*. Although not tested in this study, further consideration should be given to other pathways which regulate IL-1β processing in neutrophils. A cytosolic DNA sensor AIM2 is highly expressed in human neutrophils, in addition to NLRP3, and DNA treatment enhances the production of IL-1β in neutrophils (42). *H. pylori* DNA induces activation of TLR9 and secretion of cytokines in DCs (43). Accordingly, it is necessary to clarify whether AIM2 contributes to *H. pylori*-induced IL-1β production in neutrophils, even though it was dispensable in response to *H. pylori* in DCs for IL-1β secretion and caspase-1 processing (16). It should also be considered the involvement of extracellular neutrophil proteases, as they can lead to IL-1β processing in neutrophils through a caspase-1-independent pathway (44).

## Data Availability Statement

The raw data supporting the conclusions of this article will be made available by the authors, without undue reservation, to any qualified researcher.

## Ethics Statement

This animal study was reviewed and approved by the Institutional Animal Care and Use Committee of Chonnam National University.

## Author's Note

This manuscript has been released as a Pre-Print at Jang et al. (45).

## Author Contributions

A-RJ acquired, analyzed, and interpreted data as well as developed study concept and wrote manuscript. M-JK, J-IS, S-WK, J-YP, J-HA, T-SL, D-YK, B-GC, and M-WS contributed to part of the experiments. S-JY and M-KS wrote manuscript. J-HP developed the study concept, obtained funding and ethics, interpreted data, and wrote manuscript. All authors read and approved the final manuscript.

## Conflict of Interest

The authors declare that the research was conducted in the absence of any commercial or financial relationships that could be construed as a potential conflict of interest.
