# Supplementary material for: Unveiling the Crucial Role of Type IV Secretion System and Motility of Helicobacter pylori in IL-1β Production via NLRP3 Inflammasome Activation in Neutrophils
Source: Front Immunol. 2020 Jun 9;11:1121. doi: 10.3389/fimmu.2020.01121 (PMC7295951; doi:10.3389/fimmu.2020.01121)
Supplement: Supplementary file 1 [file Data_Sheet_1.pdf]

# Supplementary Material

## Reagent and inhibitor assay

Oxidized ATP (oxATP, Cat No. A6779), potassium chloride (Cat No. P4504), and N-acetyl-L-cysteine (NAC, Cat No. A9165) were obtained from Sigma-Aldrich (St. Louis, MO, USA). CA-074 methyl ester (CA-074Me) was purchased from Calbiochem (La Jolla, CA, USA, Cat No. 205531). The peritoneal neutrophils were infected with *H. pylori* P1WT (MOI 100) by exposure for 24 h in the absence or presence of each inhibitor for 2 h at the indicated concentrations.

## Measurement of lactate dehydrogenase (LDH)

The level of LDH in culture supernatants of *H. pylori*-infected cells was determined by using a commercial kit (CytoTox96®; Promega, Madison, WI, USA, Cat No. G1780).

## Preparation of bone-marrow-derived dendritic cells (BMDCs)

Bone marrow-derived dendritic cells (BMDCs) were isolated and differentiated as described previously [1]. Briefly, BMDCs were cultured in RPMI 1640 (Welgene, Gyeongsan, Gyeongsangbuk-do, Korea) containing 10% FBS, 1% P/S, 2 mM L-glutamine, 50 μM 2-mercaptoethanol (Sigma-Aldrich), and 20 ng/ml GM-CSF (Peprotech, Rocky Hill, NJ, USA) in a 5% $CO_2$ incubator at 37°C for 9 days. Fresh medium was added both three and six days later. These cells were seeded in 48-well plates at a density of $2\times10^5$ cells/well and incubated in a 5% $CO_2$ incubator at 37°C. Subsequently, cells were infected with *H. pylori* at the indicated dose for 24 h.

**Allelic exchange mutagenesis**

A *hp0601* (encoding FlaA) knockout mutant was generated by allelic exchange in *H. pylori* 26695. Chromosomal DNA of *H. pylori* 26695 was extracted using Primeprep Genomic DNA Extraction Kit (K-3000, Genetbio Inc., Korea). The allelic exchange fragments were constructed by amplification of a *hp0601*-upstream fragment by using primers Hp0601upF (5'-ATGGCTTTTCAGGTCAATACAAATATC-3' and Hp0601upR (5'-ATGGTTCGCTGGGTTTATCGCAATCGCTTGACCCAAACTG-3') and of a *hp0601*-downstream fragment by using primers Hp0601dwF (5'-TTACTGGATGAATTGTTTTAGTACCGCAAAATCAAATGATTAGCACCGT-3') and Hp0601dwR (5'-AAGCCTTAAGATATTTTGTTGAACGG-3'). Hp0601upR and Hp0601dwF had overlapping sequences and incorporate sequences of the kanamycin resistance gene, *aphA-3* from plasmid pIP1433-KM (underlined). Overlap PCR was performed to assemble three fragments including *hp0601*-upstream (216bps), *hp0601*-downstream (218bps), and *aphA-3* fragment (1,425bps). Briefly, the PCR amplification was performed using reaction mixture (50 μl) containing 10 pmol of each primer, 10 nmol of dNTPs, 2.5 U *pfu* DNA polymerase in 1 x *pfu* DNA polymerase reaction buffer (EBT-1011, ELPIS Biotech. Inc., Korea). The resulting construct was used for natural transformation of *H. pylori* 26695. The allelic exchange mutants were selected by plating on brucella agar supplemented with 20ug/ml kanamycin, and confirmed by DNA sequencing.

**Bacterial motility assay**

Motility analysis was performed as described previously [2] with slight modifications. Motility of bacteria assessed in semi-solid medium prepared with brucella broth containing 0.4% agar in petri plates. The plates were inoculated with bacteria by stabbing the needle of inoculum

into the soft agar, and then incubated for 5 days at 37°C under microaerobic conditions.

**Isolation of human neutrophil and bacterial infection**

Neutrophils were isolated from Single Donor Human whole blood (Innovative Research, Novi, MI, USA, Cat No. IPLA-WB1) using the EasySep$^{TM}$ Direct Human Neutrophil Isolation Kit in accordance with the manufacturer's instructions (STEMCELL Technologies, Vancouver, Canada, Cat No. 19666). Purity of isolated neutrophils was confirmed by flow cytometry (Miltenyi Biotec, Bergisch Gladbach, Germany). Isolated cells were stained with APC-conjugated anti-CD45 (BD Biosciences, San Jose, CA, USA, Cat No. 555485), FITC-conjugated anti-CD16 (BD Biosciences, Cat No. 555406), and PE-conjugated anti-CD66b (BD Biosciences, Cat No. 561650). Cell showing $CD16^+$ and $CD66b^+$ were 98.86% (Supplementary Figure 11A). To confirm whether the isolated cells show neutrophil-like morphology, the cells were centrifuged by cytospin at 700 rpm and stained with a Diff-Quik staining solution. Most cells were composed of neutrophil-like multinucleated cells (Supplementary Figure 11B). These cells were seeded into a 48-well plate ($2\times10^5$/well) and subsequently infected with *H. pylori* P1WT, P1Δ*CagL*, and P1Δ*FlaA* (MOI 100) for 24 h. The levels of IL-1β (Cat No. DY201) and TNF-α (Cat No. DY210) were measured in culture supernatants by using a commercial ELISA kit (R&D Systems, Minneapolis, MN, USA).

## References


1. Lutz MB, Kukutsch N, Ogilvie AL, Rossner S, Koch F, Romani N, et al. An advanced culture method for generating large quantities of highly pure dendritic cells from mouse bone marrow. *J Immunol Methods*. (1999) 223:77-92. doi: 10.1016/s0022-1759(98)00204-x.
2. Cerda O, Rivas A, Toledo H. *Helicobacter pylori* strain ATCC700392 encodes a methyl-accepting chemotaxis receptor protein (MCP) for arginine and sodium bicarbonate. *FEMS Microbiol Lett*. (2003) 224:175-81. doi: 10.1016/s0378-1097(03)00423-3.
