# Supplementary material for: Unveiling the Crucial Role of Type IV Secretion System and Motility of Helicobacter pylori in IL-1β Production via NLRP3 Inflammasome Activation in Neutrophils
Source: Front Immunol. 2020 Jun 9;11:1121. doi: 10.3389/fimmu.2020.01121 (PMC7295951; doi:10.3389/fimmu.2020.01121)
Supplement: Supplementary file 2 [file Data_Sheet_2.zip › Supplementary Figures/Supplementary Figure 1.docx]

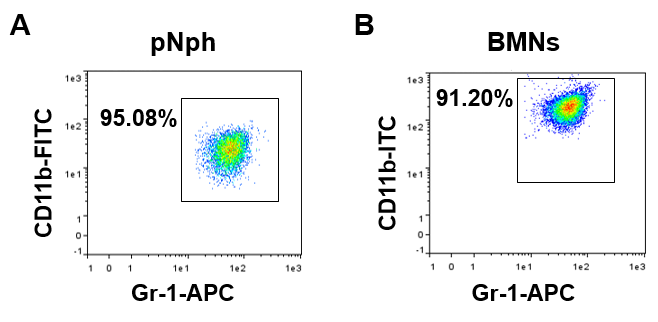


**Supplementary Figure 1. Flow cytometry analysis to determine the purity of isolated neutrophils.** Peritoneal neutrophils (A) and BMNs (B) were stained with FITC-conjugated anti-CD11b and APC-conjugated anti-Gr-1 and analyzed using flow cytometry.
