# Supplementary material for: Unveiling the Crucial Role of Type IV Secretion System and Motility of Helicobacter pylori in IL-1β Production via NLRP3 Inflammasome Activation in Neutrophils
Source: Front Immunol. 2020 Jun 9;11:1121. doi: 10.3389/fimmu.2020.01121 (PMC7295951; doi:10.3389/fimmu.2020.01121)
Supplement: Supplementary file 2 [file Data_Sheet_2.zip › Supplementary Figures/Supplementary Figure 10.docx]

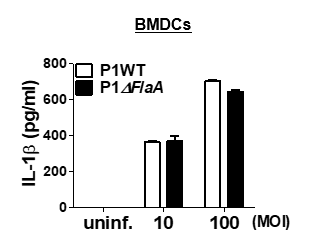


**Supplementary Figure 10. *H. pylori* flagellin is not involved in the production of IL-1β in BMDCs.** BMDCs were infected with infected with *H. pylori* P1WT and ∆*flaA* (MOI 10 and 100) for 24 h. The concentration of IL-1β in the supernatant was measured by ELISA.
