# Supplementary material for: Unveiling the Crucial Role of Type IV Secretion System and Motility of Helicobacter pylori in IL-1β Production via NLRP3 Inflammasome Activation in Neutrophils
Source: Front Immunol. 2020 Jun 9;11:1121. doi: 10.3389/fimmu.2020.01121 (PMC7295951; doi:10.3389/fimmu.2020.01121)
Supplement: Supplementary file 2 [file Data_Sheet_2.zip › Supplementary Figures/Supplementary Figure 11.docx]

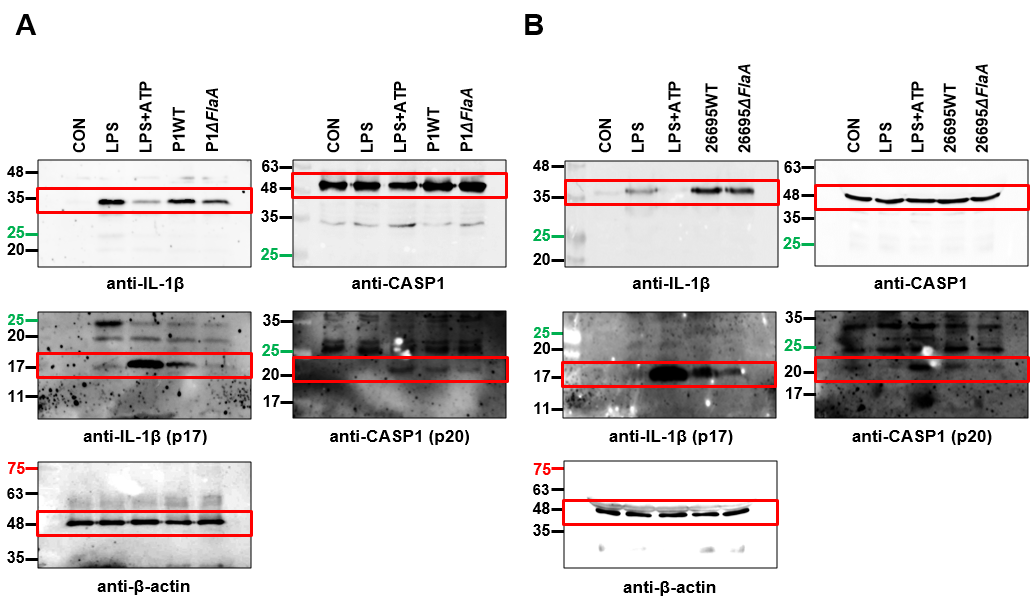


**Supplementary Figure 11. Uncropped images related to the western blots shown in Figures 5A, B.** The red boxes denote the regions of the western blots presented in the figures.
