# Supplementary material for: Unveiling the Crucial Role of Type IV Secretion System and Motility of Helicobacter pylori in IL-1β Production via NLRP3 Inflammasome Activation in Neutrophils
Source: Front Immunol. 2020 Jun 9;11:1121. doi: 10.3389/fimmu.2020.01121 (PMC7295951; doi:10.3389/fimmu.2020.01121)
Supplement: Supplementary file 2 [file Data_Sheet_2.zip › Supplementary Figures/Supplementary Figure 12.docx]

**
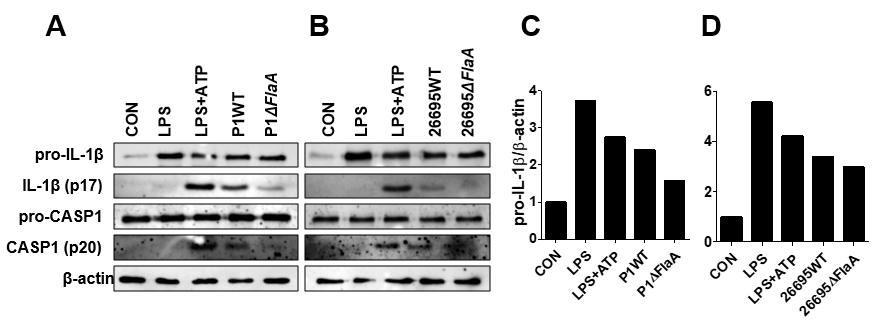
**

**Supplementary Figure 12. *H. pylori* flagellin induces activation of IL-1β and caspase-1 in response to *H. pylori* in peritoneal neutrophils.** Peritoneal neutrophils were infected with *H. pylori* P1WT and ∆*flaA* (MOI 100) for 6 h (A-D). We used culture supernatants and cell lysates to detect immature and cleaved forms of caspase-1 and IL-1β by Immunoblotting (A-D). Antibody against β-actin was used as a loading control.
