# Supplementary material for: Unveiling the Crucial Role of Type IV Secretion System and Motility of Helicobacter pylori in IL-1β Production via NLRP3 Inflammasome Activation in Neutrophils
Source: Front Immunol. 2020 Jun 9;11:1121. doi: 10.3389/fimmu.2020.01121 (PMC7295951; doi:10.3389/fimmu.2020.01121)
Supplement: Supplementary file 2 [file Data_Sheet_2.zip › Supplementary Figures/Supplementary Figure 13.docx]

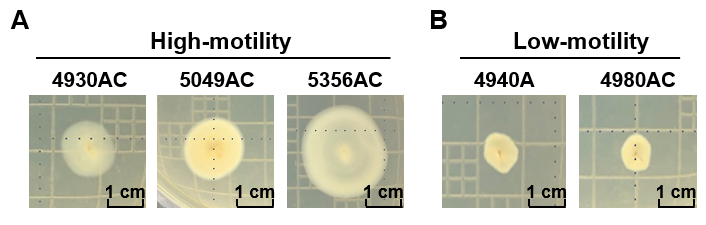


**Supplementary Figure 13. Motility test for *H. pylori* grown on semi-solid agar medium.** To test the motility of clinical isolates of *H. pylori*, Motile strains (HP 4940AC, HP 5049AC, and HP 5356AC) (A) and Non-motile strains (HP 4940A and HP 4980AC) (B) were cultured in Brucella agar with 10% horse serum for seven days at 37°C.
