# Supplementary material for: Unveiling the Crucial Role of Type IV Secretion System and Motility of Helicobacter pylori in IL-1β Production via NLRP3 Inflammasome Activation in Neutrophils
Source: Front Immunol. 2020 Jun 9;11:1121. doi: 10.3389/fimmu.2020.01121 (PMC7295951; doi:10.3389/fimmu.2020.01121)
Supplement: Supplementary file 2 [file Data_Sheet_2.zip › Supplementary Figures/Supplementary Figure 14.docx]

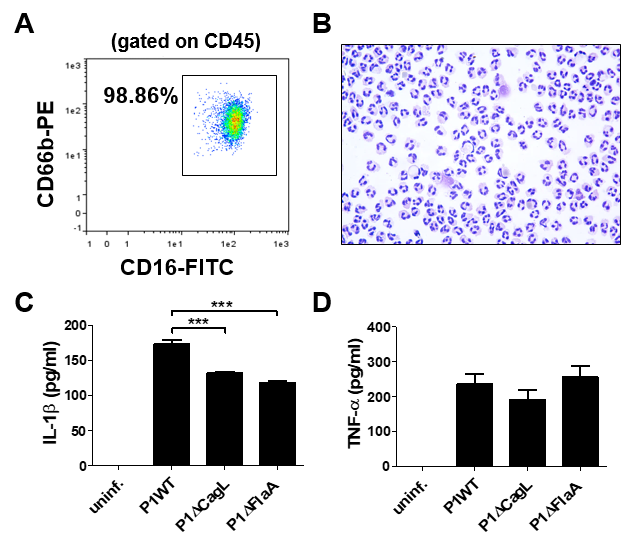


**Supplementary Figure 14. Deficiencies of bacterial T4SS and flagellin lead to impaired production of IL-1β in human neutrophils in response to *H. pylori*.** Human neutrophils isolated from single donor human whole blood were stained with APC-conjugated anti-CD45, FITC-conjugated anti-CD16, and PE-conjugated anti-CD66b, and analyzed using flow cytometry (A). Morphology of the isolated cells were confirmed by cytospin and Diff-quik staining (B). The cells were seeded in a 48-well plate (2×10^5^ cells) and infected with *H. pylori* P1WT, *∆cagL* and ∆*flaA* mutants (MOI 100) for 24 h. The concentration of IL-1β and TNF-α in culture supernatants was measured by ELISA (C and D). Results are expressed as mean ± SD. ***; *P* < 0.001.
