# Supplementary material for: Unveiling the Crucial Role of Type IV Secretion System and Motility of Helicobacter pylori in IL-1β Production via NLRP3 Inflammasome Activation in Neutrophils
Source: Front Immunol. 2020 Jun 9;11:1121. doi: 10.3389/fimmu.2020.01121 (PMC7295951; doi:10.3389/fimmu.2020.01121)
Supplement: Supplementary file 2 [file Data_Sheet_2.zip › Supplementary Figures/Supplementary Figure 3.docx]

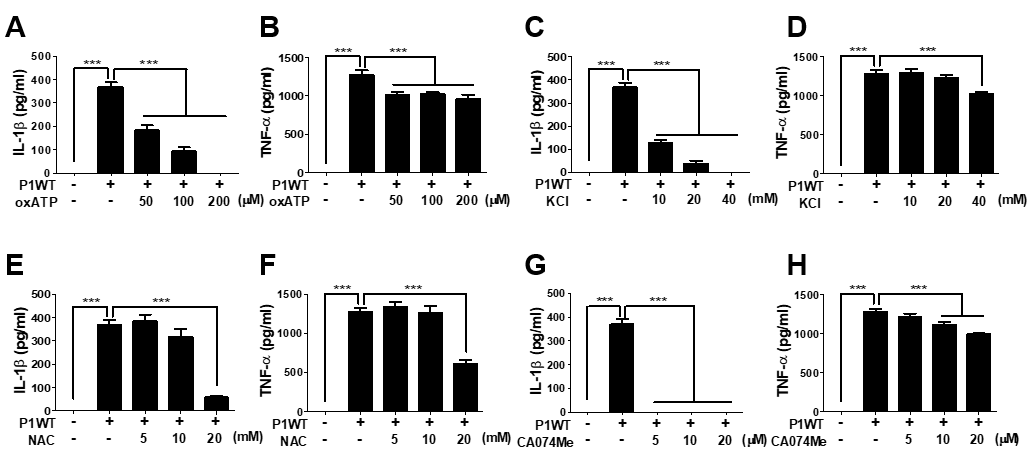


**Supplementary Figure 3. Extracellular ATP, potassium efflux, ROS production, and cathepsin B release are involved in *H. pylori*-induced IL-1β production in peritoneal neutrophils.** Peritoneal neutrophils were pretreated with the indicated concentration of oxATP (A and B), KCl (C and D), NAC (E and F), and CA-074Me (G and H) for 2 h and then cells were infected with *H. pylori* P1WT (MOI 100) for 24 h. We measured the concentration of IL-1β (A, C, E, and G) and TNF-α (B, D, F, and H) in culture in the supernatant by ELISA. Results are presented as mean ± SD. ***, *p* < 0.001.
