# Supplementary material for: Unveiling the Crucial Role of Type IV Secretion System and Motility of Helicobacter pylori in IL-1β Production via NLRP3 Inflammasome Activation in Neutrophils
Source: Front Immunol. 2020 Jun 9;11:1121. doi: 10.3389/fimmu.2020.01121 (PMC7295951; doi:10.3389/fimmu.2020.01121)
Supplement: Supplementary file 2 [file Data_Sheet_2.zip › Supplementary Figures/Supplementary Figure 4.docx]

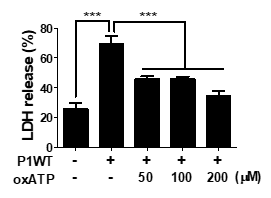


**Supplementary Figure 4. Extracellular ATP is involved in *H. pylori*-induced LDH release in peritoneal neutrophils.** Peritoneal neutrophils were pretreated with the indicated concentration of oxATP for 2 h and then cells were infected with *H. pylori* P1WT (MOI 100) for 24 h. The level of LDH was measured in the culture supernatants. The results are presented as the mean ± SD. ***, *P* < 0.001.
