# Supplementary material for: Unveiling the Crucial Role of Type IV Secretion System and Motility of Helicobacter pylori in IL-1β Production via NLRP3 Inflammasome Activation in Neutrophils
Source: Front Immunol. 2020 Jun 9;11:1121. doi: 10.3389/fimmu.2020.01121 (PMC7295951; doi:10.3389/fimmu.2020.01121)
Supplement: Supplementary file 2 [file Data_Sheet_2.zip › Supplementary Figures/Supplementary Figure 5.docx]

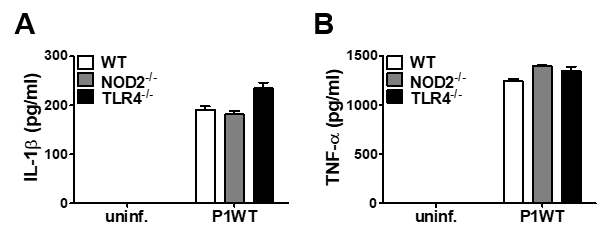


**Supplementary Figure 5. The deficiency of NOD2 and TLR4 is not involved in *H. pylori* induced production of IL-1β and TNF-α in BMNs.** WT and NOD2-, and TLR4-deficient BMNs (A and B) were infected with P1WT (MOI 100) for 24 h. The concentration of IL-1β (A) and TNF-α (B) in the supernatant was measured by ELISA.
