# Supplementary material for: Unveiling the Crucial Role of Type IV Secretion System and Motility of Helicobacter pylori in IL-1β Production via NLRP3 Inflammasome Activation in Neutrophils
Source: Front Immunol. 2020 Jun 9;11:1121. doi: 10.3389/fimmu.2020.01121 (PMC7295951; doi:10.3389/fimmu.2020.01121)
Supplement: Supplementary file 2 [file Data_Sheet_2.zip › Supplementary Figures/Supplementary Figure 6.docx]

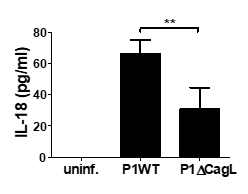


**Supplementary Figure 6. T4SS is involved in *H. pylori*-induced production of IL-18 in peritoneal neutrophils.** Peritoneal neutrophils were infected with *H. pylori* P1WT and isogenic mutant deficient in cagL (MOI 100) for 24 h. The concentration of IL-18 in supernatant was measured by ELISA. Results are presented as mean ± SD. **, *P* < 0.01.
