# Supplementary material for: Unveiling the Crucial Role of Type IV Secretion System and Motility of Helicobacter pylori in IL-1β Production via NLRP3 Inflammasome Activation in Neutrophils
Source: Front Immunol. 2020 Jun 9;11:1121. doi: 10.3389/fimmu.2020.01121 (PMC7295951; doi:10.3389/fimmu.2020.01121)
Supplement: Supplementary file 2 [file Data_Sheet_2.zip › Supplementary Figures/Supplementary Figure 8.docx]

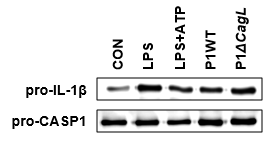


**Supplementary Figure 8. Caspase-1 and IL-1β processing is mediated *H. pylori* T4SS in mouse neutrophils.** Peritoneal neutrophils (A) were infected with P1WT and ∆*cagL* (MOI 100) for 6 h. We used culture supernatants and cell lysates to detect immature and cleaved forms of caspase-1 and IL-1β by Immunoblotting (A). Antibody against β-actin as a loading control was also used.
