# Supplementary material for: Unveiling the Crucial Role of Type IV Secretion System and Motility of Helicobacter pylori in IL-1β Production via NLRP3 Inflammasome Activation in Neutrophils
Source: Front Immunol. 2020 Jun 9;11:1121. doi: 10.3389/fimmu.2020.01121 (PMC7295951; doi:10.3389/fimmu.2020.01121)
Supplement: Supplementary file 2 [file Data_Sheet_2.zip › Supplementary Figures/Supplementary Figure 9.docx]

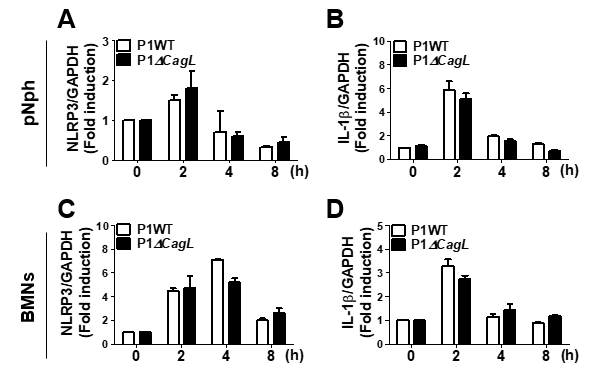


**Supplementary Figure 9. *H. pylori* T4SS not involved in pro-IL-1β processing in mouse neutrophils.** Peritoneal neutrophils (A and B) and BMNs (C and D) were infected with *H. pylori* P1WT and ∆*cagL* (MOI 100) at the indicated time points. We evaluated gene expression of NLRP3 (A and C) and IL-1β (B and D) by real-time PCR.
